# Equipped for Migrations Across High Latitude Regions? Reduced Spur Length and Outcrossing Rate in a Biennial *Halenia elliptica* (Gentianaceae) With Mixed Mating System Along a Latitude Gradient

**DOI:** 10.3389/fgene.2018.00223

**Published:** 2018-06-25

**Authors:** Ming-Liu Yang, Lin-Lin Wang, Guo-Peng Zhang, Li-Hua Meng, Yong-Ping Yang, Yuan-Wen Duan

**Affiliations:** ^1^Key Laboratory of Yunnan for Biomass Energy and Biotechnology of Environment, Key Laboratory of Ecological Adaptive Evolution and Conservation on Animals-Plants in Southwest Mountain Ecosystem of University in Yunnan Province, School of Life Sciences, Yunnan Normal University, Kunming, China; ^2^Kunming Institute of Botany, Chinese Academy of Sciences, Kunming, China; ^3^Institute of Tibetan Plateau Research at Kunming, Chinese Academy of Sciences, Kunming, China

**Keywords:** *Halenia elliptica*, spur length, mixed mating system, latitude gradient, autonomous selfing, inbreeding depression

## Abstract

*Halenia* (Gentianaceae) originated from the mountain regions of East Asia, and diversified in America following long migrations via Beringia. While *Halenia elliptica*, one species of the genus in China, migrated toward high latitudes in China. Spur length of *H. elliptica* is highly variable. We examined the relationship between spur length and mating pattern along a latitude gradient. Field experiments were performed in two populations of *H. elliptica*, and we found that this species could produce seeds via both autonomous selfing and the aid of pollinators, suggesting a mixed mating system. In seven populations of *H. elliptic*a along a latitudinal gradient, we found a trend of decrease in spur length with the increase of latitude. Based on molecular data from 11 microsatellite loci, we found that multilocus outcrossing rate decreased with the increase of latitude while the estimated inbreeding depression increased significantly, indicating that a high degree of inbreeding depression might have prevented evolution toward complete selfing in the high latitude populations with short spur length, and thus maintained mixed mating system of *H. elliptica*. Our results suggest that the mixed mating system of this species might be helpful in overcoming pollinator scarcity in newly colonized populations toward high latitudes after its origination in the mountain regions of China, and the decrease of spur length in the high latitude populations could result from reduced resource allocation to pollinator associated traits.

## Introduction

The intercontinental disjunct distribution of closely related plant species between East Asia and North America is a good resource in understanding the affinity between the biodiversity hotspot in China (The Mountains of Southwest China) and those in America, which has been of particular interest to botanists and biogeographers for a long time (Wen, 1999; Qian, 2002). Phylogenetic analyses suggested that the floristic migrations via the Beringia across the northern Pacific have been played an important role in the formation of the intercontinental geographic pattern (Wu, 1983; Qian, 2002; Wen et al., 2010). Despite of the fact that it is generally clear on formation of the intercontinental disjunct pattern of plant species between East Asia and North America (Wen et al., 2014; Chen et al., 2018), evidences on the evolution of plant reproductive systems during long distance migration are still lacking.

When plants colonize new habitats, reproductive success is often limited by mate limitation and pollinator limitation, making the evolution of sexual reproduction less dependent on animal pollinators. Thus, both autonomous selfing (Eckert et al., 2006) and abiotic pollination (Culley et al., 2002) could be favored by plant species in new habitats because these two reproductive modes could ensure reproductive success without the aid of pollinators. Recent evidence from a plant species with both insect and wind pollination suggested that wind pollination was intensified in the marginal populations due to the reduced pollinator service after range expansion (Wang et al., 2017). For plants with autonomous selfing, despite of the fact that selfing would reduce fitness of progeny via inbreeding depression and seed discounting (Herlihy and Eckert, 2002), autonomous selfing would still be selected under pollinator scarcity. Accordingly, mixed mating system would yield great reproductive assurance and ensure outcrossing under various pollination environments (Kalisz et al., 2004), and could represent a stable stage due to the high frequency (42%) in angiosperms (Goodwillie et al., 2005).

In plants with frequent selfing, resources allocated to flowers might decrease to reduce the flower structures that are associated with outcrossing or attraction to pollinators. Generally, in comparison with congeneric or intra-specific outcrosser, selfing species often have smaller and/or less flowers (Goodwillie et al., 2010), reduced herkogamy (Chen et al., 2009), and pollen:ovule ratio (Cruden, 1977). This so-called selfing syndrome has been discovered in many plant species (Sicard and Lenhard, 2011), and also has been demonstrated with the transition from outcrossing to selfing (Button et al., 2012). Nectar spur, where nectar is produced, is an important floral trait related to pollination specialization. Changes of nectar spur is generally associated with pollinator shift and the resultant speciation (Whittall and Hodges, 2007; Kramer and Hodges, 2010), and thus could be considered to be a key innovation of speciation in some plant species (Hodges and Arnold, 1995; Hodges, 1997; Sharma et al., 2014). In contrast, little is known about how spur length and the resulted mating system varied during range expansions.

*Halenia* (Gentianaceae), a genus with highly varied spur lengths, was demonstrated to originate in East Asia (probably the Himalaya-Hengduan Mountains) and migrate northward into North America via Beringia, then into South America (von Hagen and Kadereit, 2003). Although being highly varied, nectar spur was not considered to be a key innovation of the whole genus since the spur length variation within this genus from North America was not related to speciation rate (von Hagen and Kadereit, 2003), indicating that the variation of spur length could be resulted from ecological adaptations to new habitats and possible shifts of mating system. Therefore, in this paper, we performed field experiments in two populations located at different latitude sites to examine the mode of seed production of *Halenia elliptica* in natural conditions, and investigated the spur length variation in seven populations along a latitudinal gradient that could be the possible migration route toward Beringia. We also collected leaves from maternal plants and corresponding seeds to examine the variation of outcrossing rate based on SSR markers in the seven populations. Specifically, we addressed the following questions: (1) How does *H. elliptica* produce seeds in the field populations? (2) How do spur lengths vary along latitudinal gradient? (3) How do outcrossing rates vary along latitudinal gradient? (4) Are there any relationships between spur length and outcrossing rate?

## Materials and Methods

### Plant Species

*Halenia* is a large genus of Gentianaceae, but only two species of them are found in China. *Halenia corniculata* (L.) Cornaz is distributed in northern China, while *H. elliptica* D. Don has a large distribution range in China (Ho and Prigle, 1995). This genus originated in southwest China, with *H. elliptica* originating earliest in this genus while *H. corniculata* as a derivative during the migration to America via Beringia (von Hagen and Kadereit, 2003). In comparison with the other genera of Gentianaceae, *Halenia* is characterized with four spurs (Ho and Prigle, 1995). *Halenia elliptica* is a biennial herb in field conditions, and distributed widely in China. *Halenia elliptica* flowers, depending on population locations, from July to September, and each plant has many flowers (generally more than 20). Although two varieties with different flower sizes were accepted traditionally (Ho and Prigle, 1995), our former investigations based on more than 1000 specimens suggested that the two varieties should be merged since the flower sizes varied continuously (Wang et al., 2011).

### Field Experiments

In 2016, we performed field experiments to investigate seed production and self-compatibility in two *H. elliptica* populations with different latitudes (Lijiang and Huangyuan, **Table 1**). In the field, we performed the following five treatments. (1) Flowers were emasculated in the bud stage and left for free pollination to examine the seed production by pollinators. (2) Flowers were netted in the bud stage to examine the seed production by autonomous selfing. (3) Flowers were emasculated in the bud stage and hand-pollinated three times with pollen from the same plant after stigma opened to examine self-compatibility. (4) Flowers were emasculated in the bud stage and hand-pollinated three times with pollen from different plant that is more than 10 m from the receptive plant after stigma opened. (5) Flowers that were naturally pollinated were treated as control. All treatments were performed on the apical flowers on different plants, and the sample size for each treatment was more than 50. When fruits were ripe but before dehiscence, we collected the fruits and determined the seed production per fruit in laboratory. A general linear model was employed to examine the differences in seed production, with treatment and population as fixed factors in SPSS version 20.0.

**Table 1:** Population information of *Halenia elliptica.*

Pop. code	Location	No. maternal plants	No. progeny	Latitude (°)	Longitude (°)	Altitude (m)
HM12	Yunnan, Lijiang	7	33	27.00	100.2	2669
HM15	Sichuan, Kangding	12	69	30.00	101.94	3012
HM14	Sichuan, Lixian	16	92	31.62	102.86	3325
HM10	Sichuan, Hongyuan	7	40	32.79	102.5	3521
HM13	Qinghai, Banma	3	20	33.27	100.67	3926
HM11	Qinghai, Huangyuan	17	85	36.79	101.11	3005
HM16	Qinghai, Qilian	16	113	38.21	100.25	2865

### Seed Collection and Outcrossing Rate

In 2015, we collected seeds from seven field populations along a latitudinal gradient (**Table 1**). In each population, we selected 20 healthy maternal plants that were 50 m far away from each other to ensure these plants could cover the population. First, we collected fresh leaves of the selected maternal plants and kept them in silica gel. Then we collected 20 fruits randomly on each maternal plant and kept them separately in paper bags. In addition, we measured the spur lengths of 20 randomly selected flowers on different plants using a digital caliper. Linear regression analysis was performed to examine the relationships between spur length and latitude, longitude, and altitude in SPSS version 20.0.

In laboratory, seeds from one maternal plant were sowed in Petri dishes separately after being vernalized in Gibberellin GA3 with a concentration of 500 ppm for 3 days to break seed dormancy. Since the seed germination rate of *H. elliptica* was generally low in laboratory, those plants with a small sample size (less than 3) of germinated seeds were not included in the next experiments. In total, the sample size was reduced to 452 seedlings from 78 maternal plants (**Table 1**). Then the total genomic DNA from leaves of the selected maternal plants and seedling using the Plant Genomic DNA Extraction Kit (DP320-03, Tiangen, Beijing, China). DNA were then amplified with 11 microsatellite loci [HM17 (MH192924: GenBank accession number, the same in the following), HM20 (MH192927), HM22 (MH192929), HM68 (MH192949), HM85 (MH192953), HM88 (MH192956), HM103 (MH192962), HM108 (MH192965), HM115 (MH192967), HM119 (MH192968) and HM121 (MH192969)] (Ming-Liu Yang et al., unpublished). PCR were performed in a total volume of 25 mL, containing 10–40 ng (1 μL) of template DNA, 9ul 2× Power Taq PCR MasterMix, 1.75 μL (10 pmol) of each primer and 1 U of Taq DNA polymerase, and 11.5 μL Nuclease-Free Water. The PCR profile consisted of an initial denaturation step of 4 min at 94°C followed by 35 cycles of 90 s at 94°C, 50 s at an annealing temperature of 43–60°C and 50 s at 72°C, and a final extension step of 7 min at 72°C (Yin et al., 2015). The PCR products were run on an ABI 3730 automatic sequencer (Applied Biosystems) and genotypes were analyzed using GeneMapper software version 4.0 (Applied Biosystems).

Based on the 11 microsatellite loci, outcrossing rates on population level were estimated using MLTR version 3.4 (Ritland, 2002) based on 1000 bootstraps (Routley et al., 1999), through which we calculated the multilocus outcrossing rates (*t*_m_) based on the mixed mating model. Adult inbreeding coefficient (*F*) was estimated by the MLTR based on comparisons of parents and progenies. We further estimated the relative progeny fitness (ω) based on the formula ω = (2^∗^ *t*_m_^∗^ *F*)/((1-*t*_m_)^∗^(1-*F*)) (Ritland, 1990; Koelling et al., 2012), and then the inbreeding depression coefficient (δ) was calculated as 1-ω. Linear regression analysis was performed to examine the relationships between the measured variables (outcrossing rate and inbreeding depression) and latitudes, longitudes and altitudes, and the relationships between outcrossing rate and spur length and inbreeding depression in SPSS version 20.0. We also compared the inbreeding depression coefficient with the theoretically predicted threshold (0.5) (Charlesworth and Charlesworth, 1987) with one-sample test in each of the seven populations to examine the evolutionary trends toward selfing or outcrossing in each population.

## Results

### Field Experiments

**FIGURE 1:**
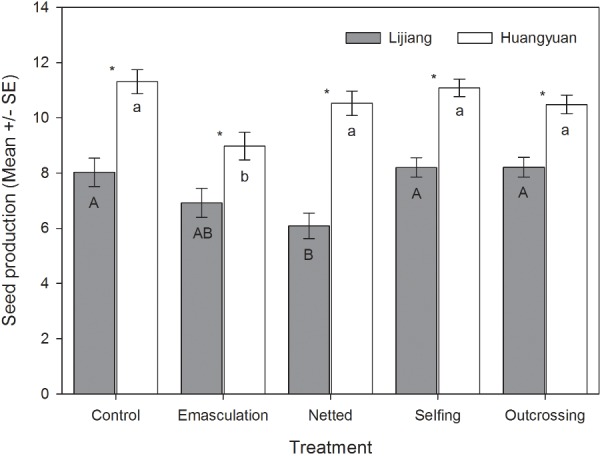
Seed production (mean ± SE) of flowers subjected to different treatments in two populations (Lijiang: lower latitude, Huangyuan: higher latitude) of *Halenia elliptica*. Control, emasculation, netted, selfing, and ourcrossing indicate naturally pollinated flowers, emasculated flowers without netting in the bud stage, netted flowers without emasculation in the bud stage, hand selfing and hand outcrossing, respectively. Values with same letters indicate that the difference is not significant at the 0.05 level among different treatments, and asterisks indicate the difference is significant at the 0.01 level between the two populations.

In the two populations with different latitudes, no difference in seed production was found between hand-selfed and hand-outcrossed flowers (**Figure 1**), indicating that *H. elliptica* was fully self-compatible. Furthermore, hand-outcrossing did not increase seed production in comparison with naturally pollinated flowers (**Figure 1**), suggesting that seed production of *H. elliptica* was not pollen limited in natural pollination environments. Seed production of flowers in the Huangyuan population at a high latitude was generally higher than that in the Lijiang population at a low latitude (**Figure 1**, *P* < 0.01 for each treatment). When flowers were netted or emasculated, seed production was reduced in both Huangyuan population and Lijiang population (**Figure 1**, *P* < 0.05), indicating that our treatments reduced seed production of *H. elliptica* to a certain degree. Collectively, in our field experiments, *H. elliptica* could produce seeds via both autonomous selfing (76–93% of naturally pollinated flowers) and the aids of pollinators (79–86% of naturally pollinated flowers), suggesting a mixed mating system of this species.

### Spur Length and Outcrossing Rate

**FIGURE 2:**
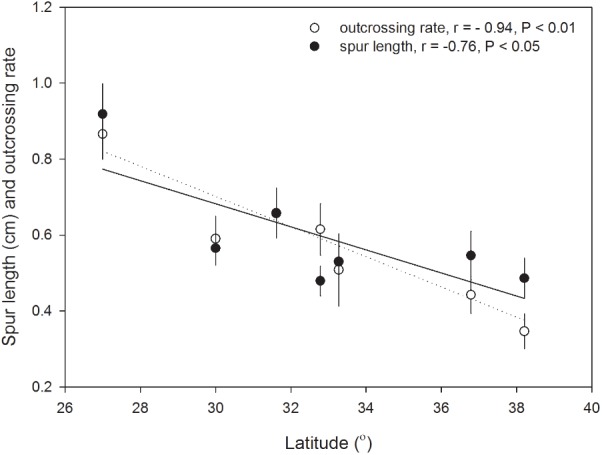
Latitudinal variations of spur length (cm, mean ± SE, open circles, and dotted line of linear regression) and outcrossing rate (mean ± SE, filled circles, and line of linear regression) in seven populations of *H. elliptica*.

Based on the measurement in the field populations of *H. elliptica*, spur length of this species ranged from 0.48 ± 0.01 to 0.92 ± 0.01 cm on population level (mean ± SE, the same in the following), and there was a significant decrease with the increase of latitude (**Figure 2**). However, the relationships between spur length and longitude (*r* = -0.25, *P* = 0.58) and altitude (*r* = -0.50, *P* = 0.26) were not significant.

**FIGURE 3:**
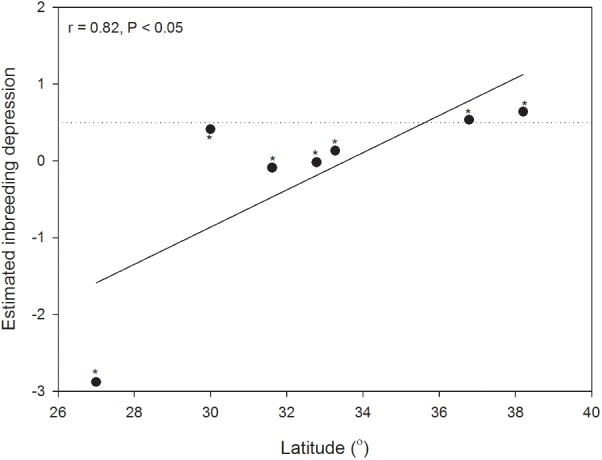
Latitudinal variations of the estimated inbreeding depression (line of linear regression) in seven populations of *H. elliptica*. Dotted line indicates the theoretically predicted threshold (0.5) below which selfing should evolve, and asterisk indicates the significant difference between the estimated inbreeding depression and the theoretically predicted threshold (0.5).

**FIGURE 4:**
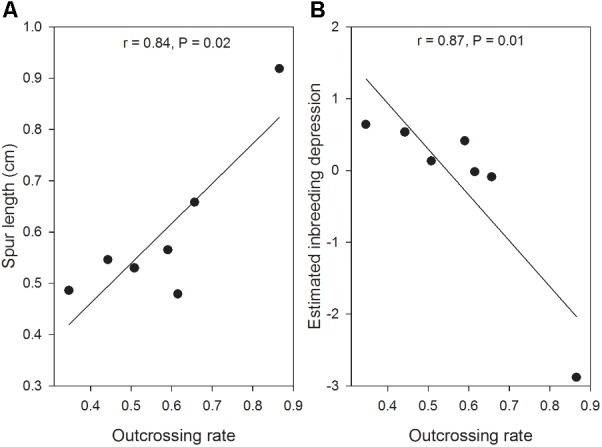
The relationships between outcrossing rate and spur length (cm, **A**) and inbreeding depression **(B)** in seven populations of *H. elliptica*.

With the results calculated from MLTR, we found that the multilocus outcrossing rate of *H. elliptica* ranged from 0.35 to 0.87 (0.57 ± 0.17) on population level, and outcrossing rate also decreased significantly with the increase of latitude (**Figure 2**). In contrast, the relationships between outcrossing rate and longitude (*r* = 0.16, *P* = 0.73) and altitude (*r* = -0.19, *P* = 0.69) were not significant. The estimated inbreeding depression (δ) ranged from -2.88 to 0.64 (-0.18 ± 1.22), and there was a trend of significant increase with the increase of latitude (**Figure 3**). No significant relationship was found between inbreeding depression and longitude (*r* = 0.32, *P* = 0.49) and altitude (*r* = 0.38, *P* = 0.40). In the seven populations, the estimated inbreeding depression in two high latitude populations was significantly higher than 0.5 in each of them, and was significantly less than 0.5 in other five populations (**Figure 3**). Additionally, we found a significant linear positive relationship between the outcrossing rate and spur length (**Figure 4A**), and a significant linear negative relationship between outcrossing rate and inbreeding depression (**Figure 4B**).

## Discussion

In this research, we performed field experiments to examine the mode of seed production of *H. elliptica*, and found that seed production of this species could be achieved with and without the aid of pollinators in natural pollination environments, indicating a mixed mating system in *H. elliptica*. In China, many other plant species in Gentianaceae have an obligate outcrossing mating system, for example, *Gentiana* (Duan et al., 2005; Hou et al., 2009), *Swertia* (Duan and Liu, 2002, 2007; Yang et al., 2007), *Megacodon* (Meng et al., 2012). Some plant species in this family have an obligate selfing mating system, for example, *Gentianopsis* (Duan et al., 2007, 2010), *Comastoma* (Zhang et al., 2011b, 2014), and *Sinoswertia* (He et al., 2013). In contrast, plant species with a mixed breeding system in Gentianaceae are rarely reported in China. Although mating system was predicted to evolve toward either predominant outcrossing or predominant selfing, depending on the degree of inbreeding depression (Lande and Schemske, 1985), a recent summary strongly suggested that a mixed mating system could be evolutionarily stable because of its wide occurrence in angiosperms (Goodwillie et al., 2005). In the populations with frequent pollinator service, predominant outcrossing would be selected, and predominant selfing would be selected to ensure reproductive assurance in populations with unreliable pollinator service. For example, in *Collinsia verna*, mixed mating depending on pollinator service would yield great reproductive assurance in unpredictable pollinator environments (Kalisz et al., 2004). In this context, plant species with mixed a mating system could colonize a larger range size than those with obligate outcrossing or selfing (Randle et al., 2009). In fact, *H. elliptica* is distributed widely in central and northern China, but many other gentians are restricted to southwest China (Ho and Prigle, 1995). The mixed mating system of *H. elliptica* might partly explain its wide distribution in China and its long distance migration toward North America via Beringia.

East Asia harbors a high biodiversity, which could be attributed to the complex topography and the diversified climates. Many plant species originate in East Asia (Chen et al., 2018) and *Halenia* also has an origin in East Asia, although its center of diversification is in America (von Hagen and Kadereit, 2003). Furthermore, *Halenia* was demonstrated to have migrated into North America via Beringia (von Hagen and Kadereit, 2003). It is hypothesized that a northward colonization might have occurred in *H. elliptica* after origination. After colonizing new inhabits, population size and density could be generally less than the core populations due to the founder effects (Hardie and Hutchings, 2010). To cope with the pollinator scarcity and the reduced mating individuals in the newly established populations, plants might lessen dependence on insect pollinators to produce seeds compared with core populations (Eckert et al., 2008; Wang et al., 2017). Our results on *H. elliptica* suggested outcrossing rate was reduced with the increase of latitude, suggesting that autonomous selfing occurred at a high possibility in the newly colonized populations with high latitudes. Furthermore, in the population at a lower latitude (**Table 1**), the estimated multilocus outcrossing rate was 0.86 (**Figure 2**), suggesting a predominant outcrossing mating system. In contrast, in the population at a high latitude (**Table 1**), the estimated multilocus outcrossing rate was 0.35 (**Figure 2**), suggesting a predominant selfing mating system. Accordingly, selfing could become dominant during northward migration of *H. elliptica* to Beringia, and this independence of insect pollinator could help this species to colonize new habitats quickly.

Evolution toward selfing depends on the degree of inbreeding depression, and it is predicted that selfing would evolve if inbreeding depression is below 0.5 (Charlesworth and Charlesworth, 1987; Holsinger, 1988). However, the degree of inbreeding depression is not constant, and could vary in different environments (Armbruster and Reed, 2005). Furthermore, continuous selfing would reduce the degree of inbreeding depression through purging the lethal recessive alleles (Husband and Schemske, 1996), which would further drive the evolution of selfing. However, we found a negative relationship between the estimated inbreeding depression and multilocus outcrossing rate in the seven populations (**Figure 4B**), indicating that inbreeding depression increased in the populations with predominant selfing at high latitudes. Furthermore, in two populations at high latitudes, the estimated inbreeding depression was more than 0.5 (**Figure 3**), suggesting the evolution toward predominant selfing has been prevented (Charlesworth and Charlesworth, 1987). This situation confirmed a “better than nothing” role of autonomous selfing in infrequent pollinator environments (Zhang et al., 2014). In addition, quantifications of pollinator visitations and the associated nectar rewards in each of the seven populations would be of great help in explaining the maintenance of mixed mating in *H. elliptica*, and we would performed these investigations in future studies.

Flowers of *H. elliptica* are characterized by one nectar spur on each of the four petals. Spur is generally considered to be a key innovation in plant speciation (Hodges and Arnold, 1995; Hodges, 1997), and a classical example on spur as a key innovation was provided by the researches on *Aquilegia*. In this genus, inter-specific spur length is highly variable, and was suggested that pollinator shift derived the evolution of long spur and the associated speciation (Whittall and Hodges, 2007). However, in *Halenia*, inter-specific variation of spur length is not generally related to the speciation rate, and thus is considered to be ecological adaptation to different environments (von Hagen and Kadereit, 2003). We found a positive relationship between spur length and outcrossing rate (**Figure 4A**) and a trend of decrease in spur length with the increase of latitude in seven populations of *H. elliptica* (**Figure 2**). These results are consistent with our investigations on herbarium of this species (Wang et al., 2011), suggesting that reduced spur length could be associated with the evolution of autonomous selfing. Nectar spur is generally associated with pollinator specialization, and continuous selfing could lead to the reduced resource allocation to floral traits that are related to outcrossing and the associated pollinator attraction (Chen et al., 2009; Goodwillie et al., 2010). Importantly, in *Halenia*, The very shortly or non-spurred species are considered to be derived from long-spurred progenitors (von Hagen and Kadereit, 2003), indicating the short spur could be a derivate trait. Furthermore, our occasional encounters in the seven populations suggested bees (including bumblebees and honeybees) should be the main pollinators of *H. elliptica.* Therefore, reduced spur lengths of *H. elliptica* in the high latitude populations could result from the reduced resource to nectar spur, which might enhance resource allocation to other flower structures due to the effects of resource reallocation (Zhang et al., 2011a,b). Our results suggested that, in the absence of pollen limitation by hand pollination, seed productions in the high latitude population were significantly higher than those in the low latitude population (**Figure 1**), which could result from the enhanced ovule number in the high latitude population, indicating the reduced resource allocation to pollinators attraction might have been reallocated to ovules. Therefore, reduced spur length of *H. elliptica* in the high latitude population could represent an adaptation to pollinator scarcity during population colonization, indicating that the spur could be an inherited trait and variations of spur length might depend on the pollinator availability.

In summary, our results suggest that *H. elliptica* has a mixed mating system, and the outcrossing rates are quite different among populations. We also found latitudinal decreases of spur length and multilocus outcrossing rate in seven populations of *H. elliptica*, suggesting the evolution of predominant selfing during its northward migration. However, the increase of inbreeding depression with the increase of latitude prevents the evolution toward complete selfing, indicating the importance of the mixed mating system in *H. elliptica*, although the reasons still remain to be explored in future studies. Our results suggest that reduced spur length in the high latitude populations might result from the reduced resource allocation to flowers associated with pollinator, and could be an adaptation to reduced pollinator services. The context-dependent pollination strategy in *H. elliptica* might partly explain its long distance migration to the high latitudes following origination in the mountain regions.

## Author Contributions

L-HM, Y-PY, and Y-WD designed the research. M-LY, L-LW, and G-PZ performed the field experiments. M-LY performed the laboratory experiments and data analysis. M-LY, L-HM, and Y-WD wrote the manuscript.

## Conflict of Interest Statement

The authors declare that the research was conducted in the absence of any commercial or financial relationships that could be construed as a potential conflict of interest.
